# Comprehensive investigation of an in-hospital transmission cluster of a symptomatic SARS-CoV-2–positive physician among patients and healthcare workers in Germany

**DOI:** 10.1017/ice.2020.268

**Published:** 2020-06-03

**Authors:** Ralph Wendt, Stephan Nagel, Olaf Nickel, Johannes Wolf, Sven Kalbitz, Thorsten Kaiser, Stephan Borte, Christoph Lübbert

**Affiliations:** 1 Department of Infectious Diseases/Tropical Medicine, Nephrology, and Rheumatology, Hospital St Georg, Leipzig, Germany; 2 Department of Pneumology, Robert-Koch-Klinik, Hospital St Georg, Leipzig, Germany; 3 Department of Laboratory Medicine and Medical Microbiology, Hospital St Georg, Leipzig, Germany; 4 Immunodeficiency Center Leipzig (IDCL) at Hospital St Georg Leipzig, Jeffrey Modell Diagnostic and Research Center for Primary Immunodeficiency Diseases, Leipzig, Germany; 5 Clinical Chemistry and Molecular Diagnostics, Institute for Laboratory Medicine, Leipzig University Hospital, Leipzig, Germany; 6 Division of Clinical Immunology, Department of Laboratory Medicine, Karolinska Institutet, Stockholm, Sweden; 7 Division of Infectious Diseases and Tropical Medicine, Department of Medicine II, Leipzig University Hospital, Leipzig, Germany; 8 Interdisciplinary Center for Infectious Diseases, Leipzig University Hospital, Leipzig, Germany

## Abstract

We investigated potential transmissions of a symptomatic SARS-CoV-2–positive physician in a tertiary-care hospital who worked for 15 cumulative hours without wearing a face mask. No in-hospital transmissions occurred, despite 254 contacts among patients and healthcare workers. In conclusion, exposed hospital staff continued work, accompanied by close clinical and virologic monitoring.

On January 27, 2020, the first infection with severe acute respiratory syndrome coronavirus 2 (SARS-CoV-2) was diagnosed in Germany.^1^ By May 20, 2020, the number of cases had increased to 176,000.^2^ To address the large number of patients at a given time, hospital capacity, especially the availability of intensive care facilities and the number of healthcare workers (HCWs), particularly doctors and nurses, are cornerstones and essential pillars in the struggle against the COVID-19 pandemic. Disease transmission among infected HCWs is a major threat that could adversely affect the capacity of hospitals to care for patients and might even endanger patients.^3^

## Case report

We report on a symptomatic SARS-CoV-2–infected physician who worked in a large 1,030-bed municipal hospital in Leipzig, Germany. At the time of the report, coronavirus disease 2019 (COVID-19) cases in Germany were rapidly increasing. The index case physician had traveled to the part of Germany with the highest COVID-19 rates at that time, thereby visiting pubs and restaurants in the city of Stuttgart (Federal State of Baden-Wuerttemberg) on March 12–13, 2020. After returning home, she felt unwell for 2 days and had a sore throat, cough, and fever.

Despite these symptoms, she went to work at the hospital without wearing a face mask or other protective devices. She remained symptomatic, particularly with subfebrile temperature and frequent coughing. On March 16, 2020, she was working an 8-hour shift in addition to a 4-hour on-call shift. She was making rounds at the hospital, caring for patients, doing admissions, discussing treatments with colleagues, having frequent contact with nurses and other healthcare staff, having lunch and coffee breaks in a small lounge area, and even sitting in a crowded lecture room along with other HCWs (Supplemental Fig. 1 online), as well as listening to employee information on the management of COVID-19 patients. During the on-call shift, she saw patients all over the hospital. The next day, she stayed at home, but she returned the following day for another 3 hours of hospital work, still coughing heavily and apparently ill. When noticed, she was immediately sent home after undergoing coronavirus testing (combined nose and throat swab), which was positive for SARS-CoV-2.

## Methods

### Laboratory setting

To assess SARS-CoV-2 infection, either Copan Liquid Amies Swabs (Copan, Brescia, Italy) or pharyngeal lavage (10 mL saline solution) was used for sampling the nasopharyngeal material of the index physician and all contacts. RNA extraction and real-time reverse-transcriptase polymerase chain reaction (RT-PCR) was performed as described in the Supplemental Material (online).

To further investigate potentially missed transmissions, we attempted to detect IgA and IgG antibodies against SARS-CoV-2 in sera, withdrawn on days 15 or16 and 22 or 23 after exposure, by an in vitro diagnostic labeled anti–SARS-CoV-2 enzyme-linked immunosorbent assay (ELISA, Euroimmun, Lübeck, Germany), following the manufacturer’s instructions.

### Statistical analysis

Only descriptive statistics were applied. Numerical variables were summarized as means, and categorical variables were given as frequencies or proportions.

### Ethical approval

Ethical approval was not required for this study because only anonymous aggregated data were used, and no medical interventions were made on human subjects. Sampling of HCWs or patients was part of hospital policy.

## Results

We identified 187 contacts with HCWs and 67 contacts with patients. Of these, 23 were identified as high-risk contacts, as defined by the World Health Organization guidance document on COVID-19 global surveillance.^4^ Table 1 summarizes the characteristics of each high-risk contact.

**Table 1.** Characteristics of High-Risk Contacts

No.	Occupation	High-Risk Contact	Personal Protective Equipment	First SARS-CoV-2 RT- PCR	Second SARS-CoV-2 RT- PCR	SARS-CoV-2 serology (IgA/IgG)
1–5	Nurse	>15 min face-to-face contact in the pneumology ward	None (eg, no face mask)	Day 5	Day 10	Days 16, 22
6	Patient	Transfer in an ambulance, 45 min drive	None (eg, no face mask)	Day 5	Day 10	Day 12
7–10	Medical technician	Sitting in the row behind the index physician for 45 min in a lecture	None (eg, no face mask)	Day 5	Day 10	Days 15, 16, 22
11	Physician					
12–13	Physician	>15 min face-to-face conversation, handover of a patient at the urology department	None (eg, no face mask)	Day 5	Day 10	Days 15, 16, 22
14	Physician	Working together with the index physician for 8 h at the same workplace, sharing lunch and sitting close together during the lecture	None (eg, no face mask)	Day 5	Day 10	Days 15, 22
15	Physician	>15 min face-to-face conversation during on-call duty	None (eg, no face mask)	Day 5	Day 10	Days 15, 22
16	Physician	Supervisor of the index physician, cumulative 30 min face-to-face discussion	None (eg, no face mask)	Day 5	Day 10	Days 15, 22
17–22	Physician	2×30 min together in the break room for lunch and coffee, room size <10 m^2^	None (eg, no face mask)	Day 5	Day 10	Days 15, 22
23	Physician	30 min face-to-face discussion	None (eg, no face mask)	Day 5	Day 10	Days 15, 22

Note. RT-PCR, reverse-transcriptase polymerase chain reaction.

All high-risk contacts were subject to active symptom-monitoring and committed to wearing a face mask during work. We tested all 254 potential contacts of the symptomatic SARS-CoV-2--positive index physician, including 67 patients, and 187 nurses and doctors, technical and medical assistants, and other healthcare staff, on day 5 after the exposure by specific RT-PCR from nose and throat swabs or pharyngeal lavage, irrespective of reported symptoms. Of 187 tested HCWs, 30 (16%) reported minor unspecific symptoms of upper airway infection (sore throat, coughing, sniffing). All tested persons turned out to be SARS-CoV-2 negative.

The 23 high-risk contacts were investigated again 10 days after exposure by specific RT-PCR from nose and throat swabs. Test results were negative, again. Additionally, all high-risk contacts and the index physician were examined serologically on days 15 or 16 and days 22 or 23 after exposure. Despite some IgA positive-to-inconclusive ratios, none showed positivity for SARS-CoV-2 IgG antibodies at follow-up except the index physician featuring seroconversion (Table 2).

**Table 2.** SARS-CoV-2 Serology Testing of High-Risk Contacts^a^ with Inconclusive (≥0.8 and <1.1) or Positive (≥1.1) Results^a^

Contact No.	Day 10	Days 15–16		Days 22–23	
		ELISA		ELISA	
	SARS-CoV-2 RT-PCR	IgA	IgG	IgA	IgG
11	Negative	**1.69**	0.247	**1.256**	0.331
14	Negative	1.097	0.113	1.087	0.167
19	Negative	0.854	0.226	0.839	0.264
23	Negative	**1.706**	0.153	**1.558**	0.183
Index	**Positive**	**2.659**	0.647	**1.732**	**1.525**

Note. RT-PCR, reverse-transcriptase polymerase chain reaction; ELISA, enzyme-linked immunoassay.

a Numbers were taken from Table 1. Test kits were obtained from Euroimmun, Lübeck, Germany. The index physician tested positive for SARS-CoV-2. Serological test results from the index physician were obtained on days 16 and 23.

## Discussion

We tested a large number of possible contact persons of a symptomatic SARS-CoV-2–infected physician among HCWs and patients on day 5 after exposure; all were negative. After a comprehensive investigation of all contact clusters, we identified 23 high-risk contacts (22 HCWs and 1 patient) and tested them again on day 10 after exposure. All RT-PCR tests remained negative for SARS-CoV-2, confirming that there was no transmission of the virus.

Extensive investigation and testing were performed because viral shedding of SARS-CoV-2 has been shown in completely asymptomatic individuals, prompting the hypothesis that clinical status is not reliable for triage and further testing.^5^ SARS-CoV-2 has frequently been detected in asymptomatic carriers, for instance, during a cruise ship outbreak in which most of the passengers and staff were tested irrespective of symptoms: 51% of the laboratory-confirmed cases were asymptomatic at the time of confirmation.^6^

For further analysis and confirmation of our results, we investigated the serum of all high-risk contacts (n = 23) on days 15 or 16 and 22 or 23 for SARS-CoV-2–specific antibodies. We found positive IgA antibodies at both times but no IgG antibodies, confirming the RT-PCR results of zero transmission. The specificities for IgA and IgG against SARS-CoV-2 were 91.3% and 100%, respectively. Although the calculated performance values were obtained in a small study cohort (n = 24), the specificities were similar to those reported in a previous study and in accordance with the manufacturer’s specifications.^7^

These results are unexpected. Considering an active SARS-CoV-2 transmission source with a presumably high viral burden and many high-risk contacts inside a hospital, massive spread was anticipated, particularly since a protective face mask was not in use. SARS-CoV-2 has been shown to persist (at least under experimental circumstances) for up to 72 hours depending on the surface type.^8^ In hospitals, surfaces are frequently cleaned and disinfected, and all HCWs reported regular handwashing, disinfection, and strict adherence to hygiene rules. Recently, the importance of presymptomatic transmission (R_p_) has been stressed (R_p_ = 0.9 of an R_0_ of 2), and the proportion of symptomatic transmission (R_s_) to the basic reproduction number R_0_ was calculated to be only 0.8 of an R_0_ of 2.^9^

A low percentage of transmission to high-risk contacts (5%) has been reported in nonhousehold members.^10^ Another study in the United States investigated the high-risk contacts of a patient among healthcare personnel (n = 32) and did not find any transmission, confirming our results. However, testing was only done in symptomatic persons after clinical monitoring, and asymptomatic transmission could have been missed.^11^ Importantly, not every infected person with SARS-CoV-2 is a super spreader, and not every infected individual in a closed room triggers a super-spreading event, although this situation has the potential to do so and therefore must be dealt with as such.^12^

In this context, our data support the recommendation to keep high-risk contacts among the hospital staff at work (especially in these difficult times with personnel shortages) when strictly using a protective mask, accompanied by close clinical and virologic monitoring.

## Supplementary material

For supplementary material accompanying this paper visit http://dx.doi.org/10.1017/ice.2020.268. click here to view supplementary material
