# Supplementary material for: Comprehensive investigation of an in-hospital transmission cluster of a symptomatic SARS-CoV-2–positive physician among patients and healthcare workers in Germany
Source: Infect Control Hosp Epidemiol. 2020 Jun 3:1–3. doi: 10.1017/ice.2020.268 (PMC7298077; doi:10.1017/ice.2020.268)
Supplement: Supplementary file 1 [file S0899823X20002688sup001.docx]

**Supplemental Figure 1:** Schematic of the lecture room. In red: SARS-CoV-2-infected index physician. In yellow: positions of high-risk contacts. In gray: empty seats. In green: moderate or low-risk contacts.


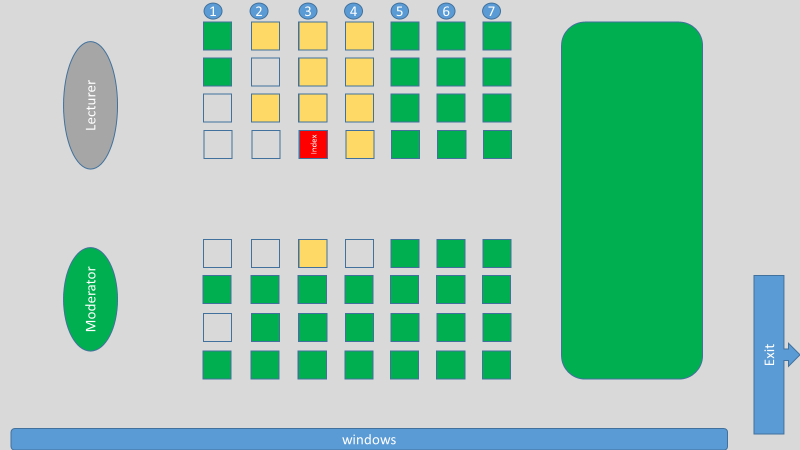


# Methods

**Performance of RNA extraction and real-time RT-PCR for SARS-CoV-2**

RNA extraction was performed on a MagNA Pure 24 System (Roche, Mannheim, Germany) and real-time RT-PCR was conducted using the LightCycler Multiplex RNA Virus Master Mix on a Lightcycler 480 RT system (both Roche, Mannheim, Germany) or Vii A7 system (Applied Biosystems, [Foster City,](https://www.bing.com/search?q=Foster+City&filters=ufn%3a%22Foster+City%22+sid%3a%22363e8f31-ef04-5ae5-0739-21b9b5368457%22&FORM=SNAPST) California, USA).

We used the Sarbecovirus specific LightMix Modular SARS-CoV (COVID-19) E gene (TIB Molbiol, Berlin, Germany) as screening assay. Positive-screened samples were confirmed by the SARS-CoV-2 specific LightMix Modular SARS-CoV-2 (COVID-19) RdRP assay (TIB Molbiol, Berlin, Germany). The E gene assay was validated with the E gene control (in vitro transcribed E gene RNA), a SARS-CoV-1 strain Frankfurt 1 RNA control, and a SARS-CoV-2 strain RNA control, all provided by the European Virus Archive GLOBAL (EVAg). For the 3 controls, the assay showed a sensitivity of 50 copies per PCR-setup. Samples with a crossing point (Cp) <36 were considered positive. The RdRP gene assay was validated with the SARS-CoV-2 RNA control (EVAg) with a detection limit of 500 copies per PCR setup. Samples with a Cp <39 were considered positive. EAV extraction controls (TIB Molbiol, Berlin, Germany) were used as internal PCR control according to the manufacturer’s instructions.
